# Effect of five lactic acid bacteria on the flavor quality of fermented sweet potato juice

**DOI:** 10.1016/j.fochx.2024.102023

**Published:** 2024-11-20

**Authors:** Bin Liang, Xue Bai, Yunfan Wang, Xiaohe Li, Yanhui Kong, Xiulian Li, Xiangquan Zeng, Wenli Liu, Huamin Li, Shuyang Sun, Hansheng Gong, Xinguang Fan

**Affiliations:** a School of Food Engineering, Yantai Key Laboratory of Nanoscience and Technology for Prepared Food, Yantai Engineering Research Center of Food Green Processing and Quality Control, Ludong University, Yantai, Shandong 264025, PR China; b School of Health, YanTai Nanshan University, Longkou, Shandong 265713, PR China; c Yantai Landscape Construction and Maintenance Center, Yantai, Shandong 264000, PR China; d School of Pharmacy, Binzhou Medical University, Yantai, Shandong 264003, PR China; e Department of Food Science, College of Agriculture, Purdue University, West Lafayette, IN 47906, USA

**Keywords:** Sweet potato, Lactic acid bacteria, Aroma profile, GC-IMS, GC–MS

## Abstract

The purpose of this research was to assess the impact of 5 lactic acid bacteria (LAB), Lactiplantibacillus plantarum, Lacticaseibacillus casei, *Streptococcus thermophilus*, Lacticaseibacillus rhamnosus and *Lactobacillus delbrueckii* subsp. bulgaricus on the characteristic flavor of the fermented sweet potato juice. Following the fermentation process, significant variations were observed in the concentrations of sugars, organic acids, as well as the overall volatile aroma compounds. LAB can effectively facilitate the production of volatile organic compounds (VOCs), including acids and ketones, thereby enhancing the aroma quality. Inoculation fermentation by LAB decreased the sweet and nutty odor, and increased fresh, floral, and citrus aroma of the sweet potato juice. The sweet potato juice fermented by 5 lactic acid bacteria strains had different flavor features, while the sample of Lp10 showed the highest overall acceptability. Compared to other strains, *L. plantarum* exerted a more significant influence on the volatile compounds present in fermented sweet potato juice.

## Highlights

• LAB could promote the VOCs in sweet potato juice, including acids and ketones.
• LAB decreased sweet, fatty, nutty odor, and increased fresh, floral, citrus aroma.
• Lp10 could produce mannitol, giving a more refreshing taste to the sweet potato juice.
• *L. plantarum* could be used as a potential starter culture for sweet potato juice.

## 1 Introduction

Sweet potato (*Ipomoea Batatas L.*) is recognized as a vital food crop globally, particularly in terms of human consumption (Nguyen et al., 2021). Sweet potatoes are widely cultivated around the world, Asia and Africa account for 85 % of global production, while the output of tubers was 29.7739 million tons (Niu et al., 2019). In China, sweet potatoes have a diverse range of applications, encompassing various sectors including culinary usage, processed foods, starch production, and animal feed. Sweet potato boasts an abundance of starch, dietary fiber, carotene, vitamins A, C, and E, as well as B vitamins. Furthermore, it is a rich source of minerals including calcium, iron, selenium, and potassium (Mashitoa et al., 2023). Sweet potato pulp contains fermentable sugars (glucose, fructose and sucrose) which are twice of common potato (de Albuquerque, Sampaio, & de Souza, 2019). The presence of sufficient fermentable sugars provides an opportunity for using fermentation as a potential method for developing higher value products from sweet potato juice.

“Yanshu No. 25” is a new sweet potato variety developed and bred by Yantai Academy of Agricultural Sciences in Shandong Province, China. “Yanshu No. 25”, which passed the national identification and Shandong provincial examination, is a new edible variety with high quality, high yield and good disease resistance. Compared with other sweet potatoes, “Yanshu No. 25” is more sweet and acceptable after roasting (Hou, Mu, Ma, & Blecker, 2020). Based on Gray relational analysis, Yanshu No. 25 demonstrated superior comprehensive nutritional values (Hou, Mu, Ma, Blecker, & Sun, 2019). Sweet potatoes have a high yield, but their quality varies. Some too large or too small sweet potatoes are not suitable for fresh consumption and need to be further processed.

Lactic acid bacteria (LAB) are a type of gram-positive bacteria that do not produce spores. They can produce a variety of metabolites and convert carbohydrate substrates into organic acids (Sato, 2023; Stabnikova, Khonkiv, Kovshar, & Stabnikov, 2023). Fermentation of various food stuffs by LAB is one of the oldest forms of food biopreservation (Zhao et al., 2023). In order to provide a direction for the high-value utilization of sweet potato, it is of great significance to the production and processing of sweet potato industry by LAB fermentation, and the changes of flavor substances and functional characteristics after fermentation need to be further studied (Bangar, Suri, Trif, & Ozogul, 2022). Different LAB have different metabolic characteristics. Compared with homo-fermentation, hetero-fermentation LAB can produce more metabolic products, such as ethanol, CO_2_, and mannitol, while having fewer acids (Moon, Kim, & Chang, 2018). Hetero-fermentation LAB can produce mannitol, ethanol and other flavor substances, giving fermented products better flavor and taste, so hetero-fermentative LABs are considered to be better vegetable pickles fermentation strains (Kim, Lee, Lee, Roh, & Kim, 2019). The use of different LAB during the fermentation process may affect the flavor characteristics of sweet potato juice products. The current presumption draws support from Tian, Shen, Yu, He, and Chen (2017), indicating that the influence of distinct LAB during fermentation holds significant impact on determining the flavor signatures of yogurt. To test this hypothesis, the present study compared the effects of different LAB on the flavor characteristics of fermented sweet potato juice.

In this research, quantitative descriptive analysis (QDA) was used to assess the sensory qualities and taste characteristics in order to examine the impact of various LAB inoculations on the fermentation of sweet potato juice. Sophisticated analytical techniques, including high performance liquid chromatography (HPLC) and amino acid automated analyzer, were employed to identify non-volatile organic compounds (non-VOCs) such as sugars, organic acids, and free amino acids (FAAs) that contribute to the taste and overall quality of fermented sweet potato juice. The fermented sweet potato juice was subjected to precise determination of volatile organic compounds (VOCs).The data from this study will be a useful resource for using LAB to manufacture sweet potato juice that has undergone fermentation. It will also give the theoretical framework for choosing an appropriate starter to create fermented sweet potato juice with desirable organoleptic qualities.

## 2 Material and methods

### 2.1 Materials and reagents

“Yanshu No. 25” sweet potatoes were obtained from Yantai Academy of Agriculture and Forestry Sciences (Yantai, Shandong Province, China). 3-Nonone (≥99 %) was used as an internal standard. The standards of sugar, organic acid, and amino acid were acquired from Aladdin (Shanghai, China). Other reagents utilized were of analytical grade and were obtained from Aladdin (Shanghai, China).

Lactiplantibacillus plantarum LD.0010 (Lp10), Lacticaseibacillus casei LD.0470 (Lc), Lactiplantibacillus plantarum LD.0001 (Lp01), *Streptococcus thermophilus* and L. *delbrueckii* subsp. bulgaricus (St-Ld) and Lacticaseibacillus rhamnosus GG® (Lr) were used for fermentation. St-Ld was purchased from Zhengzhou Yike Shangyuan Biotechnology Co., LTD., China. Lr was purchased from CHR. Hansen (Beijing) TRADING Co., LTD., China. Lp10, Lc, Lp01 were isolated and purified from Yantai sweet cherry, and are now preserved in the College of Food Engineering, Ludong University.

### 2.2 Preparation of fermented sweet potato juice

#### 2.2.1 Preparation of sweet potato juice

Fresh sweet potatoes were selected, washed, and roasted at 230 °C for 45–50 min to obtain roasted sweet potatoes. After the peeling process, the roasted sweet potatoes were cut into appropriate sizes and mixed with water at a ratio of 1:3 (sweet potato to water). The juice was ground by colloid, mixed and milled by homogenizer, sterilized, bottled and refrigerated for fermentation.

#### 2.2.2 Activation and preservation of LAB strains

The strains were stored at −80 °C and reactivated in MRS broth for 3 times at 37 °C by passing culture at 1 % (*v*/v) inoculation rate. All the strains were incubated in MRS broth at a constant temperature of 37 °C for a duration ranging from 12 to 18 h, under identical experimental circumstances. Subsequently, the cells were gathered through centrifugation at 2700 rpm for 20 min at 4 °C. Following centrifugation, the cells were washed twice with 0.85 % (*w*/*v*) saline solution to achieve a ultimate concentration of 9.0 log CFU/mL. The cells, having been resuspended, were subsequently incorporated into the sweet potato juice.

#### 2.2.3 Preparation of sweet potato juice fermented by LAB

The initial pH of unfermented sweet potato juice was 6.0, which was suitable for the growth of the vast majority of LAB. The control group of sweet potato juice without starter culture was named CK. The sweet potato juice were processed by inoculation with Lp10, Lc, Lp01, St-Ld and Lr as starters. The samples were sterilized and inoculated with 5 % (*v*/v) inoculation rate at 37 °C. To evaluate the differences among the samples, pH 4.5 was selected as the fermentation end point. The resulting samples were used for subsequent experiments.

### 2.3 HPLC analysis of sugars and organic acids

The mixture of 1 mL deionized water and 1 mL sweet potato juice (1:1, V / V) was shocked by microwave for 30 min, and centrifuged at 10000 rpm for 15 min at 4 °C to obtain the supernatant. Subsequently, these supernatants were filtered through 0.22 μm filters and dispensed into HPLC sample vials. By comparing the retention time and peak area of substrates and products with those of standard substances, the substrates and products were identified and quantified. Sugars and organic acids were analyzed by previous method (Lamani, Anu-Appaiah, Murthy, Dewir, & Rikisahedew, 2022) with some modifications.

HPLC (Shimadzu Corp, Kyoto, Japan) was used to analyze the free sugars contents in sweet potato juice with a refractive index detector (RID-10 A). Chromatographic analysis was achieved using a Shodex Asahipak NH2P-50 4E column (250 × 4.6 mm I.D., Showa Denko, Tokyo, Japan) maintained at 30 °C. The mobile phase consisted of 25 % water and 75 % acetonitrile with a flow rate set at 1 mL/min. The injection volume was10 μL.

The organic acids in sweet potato juice were carried out by HPLC equipped with a HydrospHere C18 column (250 × 4.6 mm, 5 μm) maintained at 20 °C. The mobile phase was KHPO_4_ (0.02 M, pH 3.0). The injection volume of both the sample and standard is 10 μL. Perform chromatographic separation at a flow rate of 1.0 mL/min.The diode array detector (DAD) was used to detect the organic acids at 210 nm.

### 2.4 Amino acid analyzer detection of FAAs composition

The composition and content of FAAs were determined using amino acid analyzer as previously described with some modification (Bai et al., 2022). Samples were mixed with 5 % TFA and centrifuged at 12,000 rpm for 10 min at 4 °C to remove proteins. The supernatant was filtered through a 0.22 μm nylon filter and then measured using an automatic amino acid analyzer (Hitachi L-8800, Hitachi, Tokyo, Japan) equipped with an ion-exchange resin 2622 column (4.6 mm × 60 mm, 3 μm), and a UV detector (570 and 440 nm).

### 2.5 VOCs analysis

Determination of VOCs in sweet potato juice was conducted through headspace gas chromatography-ion mobility spectrometry (HS-GC-IMS) and headspace solid phase microextraction-gas chromatography–mass spectrometry (HS-SPME-GC–MS) methods.

HS-GC-IMS instrument was equipped with the FS-SE-54-CB-1 column (15 m × 0.53 mm ID: 0.53 μm film thickness, RESTEK). A 3 mL sample was introduced into a 20 mL headspace glass vial. Following incubation at a controlled temperature of 65 °C for 15 min, 500 μL of headspace gas was injected using a heated syringe maintained at 70 °C in splitless mode. The flow rate of carrier gas in the drift tube was 150 mL/min. The initial carrier gas flow rate was 2 mL/min, held for 2 min, raised to 100 mL/min and held for 20 min. The retention index (RI) of each compound was calculated using n-ketones C4-C9 (Sinopharm Chemical Reagent Beijing Co., Ltd., China) as external references. The difference data of VOCs between samples were obtained by laboratory analysis (LAV) software in positive mode, and identified according to the retention index (RI) and drift time (DT) with the HS-GC-IMS library.

(1) $$\mathrm{Ci}\left(\mathrm{\mu g}/\mathrm{kg}\right)=\frac{\mathrm{Ai}*\mathrm{Cs}}{\mathrm{As}}$$

where Ci and Ai represent the concentration and peak area of a component; Cs and As are the concentration and peak area of 3-Nonanone. The results were expressed as μg/kg of 3-Nonanone equivalent.

HS-SPME-GC–MS analysis was performed following the method of R. Zhang, Tang, Jiang, Mo, and Wang (2021) with a slight modification on an GC 2030 series gas chromatograph coupled with an SHIMADZU GCMS-QP2020NX series mass selective detector. The capillary column was a SH stabilwax column (60 m × 0.25 mm ID, 0.25 μm). Briefly, the sample (2 mL) was placed into a 15 mL headspace vial by adding 2.5 μL of 3-nonanone (0.01 mg/mL, internal standard). Following the establishment of equilibrium conditions at 65 °C and 450 rpm for a duration of 10 min, the VOCs were allowed to adsorb onto the SPME fiber for a period of 30 min.

Subsequently, the SPME fiber underwent thermal desorption at a temperature of 250 °C for a duration of 5 min within the splitless injection port of the gas chromatograph for analytical purposes.

High-purity helium was used as a carrier gas at a flow rate of 1 mL/min. Simply put, the oven temperature was first kept at 40 °C for 3 min, heated at a rate of 5 °C/min to 115 °C, then heated at a rate of 2 °C/min to 150 °C, and finally heated at a rate of 7 °C/min to 230 °C and held for 10 min. The ionization voltage was 70 eV, and the scanning range was 50–550 *m*/*z*. The quantification of VOCs relied on a semi-quantitative approach, utilizing measurements of relative peak areas between identified compounds and the internal standard as the basis for determination.

### 2.6 Odor and taste activity determination

Odor descriptions were obtained primarily from the Perflavory website (http://www.perflavory.com/). The odor activity value (OAV) was calculated according to the ratio of the concentration of each VOCs to odor thresholds (OTs). OTs were obtained from the software1 - Flavor-Base10 Demo and valued in water. Analogously, the taste activity values (TAVs) for each FAA were calculated through the utilization of taste thresholds (TTs), TTs were gained from the literature Xiao et al. (2022).

### 2.7 Sensory evaluation

Sensory qualities were assessed through QDA based on previous studies Hou et al. (2020) with some modifications including 4 indicators: appearance (texture and gloss), flavor (sweet potato, sweet, baked, fruity and sour), taste (sour, sweet and fresh) and overall acceptability. Trained team members (including 6 males and 5 females, 20–40 years old) were recruited from the Fruit and Vegetable Preservation Laboratory of Ludong University.

The samples were presented to the group at random and were randomly numbered with three digits. The sensory evaluation was assessed utilizing a 9-point scale.

### 2.8 Statistical analysis

Experimental data were analyzed statistically using IBM SPSS Statistics 26 (IBM Corp., Armonk, NY, USA). A value of *p <* 0.05 was considered statistically significant. The partial least squares discriminant analysis (PLS-DA) was conducted utilizing the MetaboAnalyst version 5.0. Heatmap and cluster analysis were performed by TBtools version 1.108.

## 3 Results and discussion

### 3.1 Profiling of non-VOCs

#### 3.1.1 Sugars detected by HPLC

The soluble sugar content of the CK, Lp10, Lp01, Lc, St-Ld, and Lr groups, as detailed in Table S1, amounted to 55.23, 29.01, 32.32, 39.22, 20.51, and 39.20 mg/mL, respectively. Fructose, glucose, sucrose and maltose were found in each group, and mannitol was only detected in the Lp10 group. After fermentation, the free sugar content in the five fermented sweet potato juices decreased significantly, among which St-Ld decreased the most (55.23–20.51 mg/mL). The reduction in total sugars is due to the fact that LAB can convert carbohydrates such as sucrose, fructose, and glucose into organic acids (Okoye et al., 2023).

Maltose was the main sugar component in roasted sweet potato juice, which was in agreement with Hou et al. (2020) and it can be used sparingly as a mild sweetener for foods. Mannitol, a natural six‑carbon sugar alcohol, is an important raw material affecting umami and freshness taste. The fructose-mannitol cycle serves as the primary metabolic route for the biosynthesis of mannitol, and with the help of nicotinamide adenine dinucleoitde phosphate (NADPH), fructose can be catalyzed by mannitol dehydrogense (MDH) to mannitol (Alexzman, Ibrahim, & Annuar, 2023). Compared with other samples, the sample fermented with Lp10 consumed the most fructose in the juice. Additionally, it was the only sample to produce mannitol, attributed to the unique heterologous fermentation characteristics exhibited by L. *plantarum* LD.0010.

#### 3.1.2 Organic acids detected by HPLC

As indicated in Table S2, 4 distinct organic acids were identified in the samples, namely lactic acid, malic acid, citric acid, and fumaric acid. Citric acid is the main organic acid in sweet potato juice, which plays a seasoning role and produces a slight sour taste. The citric acid content before fermentation was 429.96 mg/L, and the concentration of citric acid in all samples significantly decreased after fermentation.

During the LAB fermentation process, the primary mechanism for lactic acid synthesis involves the conversion of malic acid, as well as the transformation of pyruvic acid, which is generated via the Embden-Meyerhof pathway (EMP) (H. Zhang et al., 2008). The concentration increased dramatically throughout fermentation, an increase of over 100 times due to lactobacillus consuming sugars and producing large amounts of lactic acid during fermentation. Lactic acid serves as the primary factor influencing the sour taste of fermented sweet potato juice, while malic acid is a flavor mixture that creates a strong and sharp taste. Overall, the production of lactic acid is closely related to the reduction of malic acid, indicating that LAB undergoes malic and lactic acid fermentation during the sweet potato juice fermentation process. Due to the presence of malolactic bacteria, the conversion of malic acid to lactic acid may be a decarboxylation reaction (Chen et al., 2023). This process effectively reduced the high acidity in sweet potato juice, making the final product taste softer and no longer overly stimulating, enhancing the eating experience.

Except for lactic acid, the contents of other organic acids exhibited basically the same trend of change, demonstrating a decline in their concentrations following the fermentation process. After fermentation, a significant increase in lactic acid content was observed across all samples. Concurrently, samples containing LAB exhibited a statistically significant decrease (*p* < 0.05) in the concentrations of malic acid, citric acid, and fumaric acid. Among them, the lactic acid concentration of Lr inoculated samples was the highest, which was 50,104.44 mg/L.

#### 3.1.3 FAAs detected by amino acid analyzer

As demonstrated in Table 1, nine FAAs were detected across all samples, with three of them being designated as essential FAAs (EAAs). The majority of FAAs have been reported to be generated by LAB through proteolytic enzymes and protein degradation mechanisms and they mainly present sour/umami (Asp, Glu), sweet (Thr, Ser, Gly, Ala), and bitter (Val, Lys, His) taste (León-Vaz et al., 2023). The total TAVs of the fermented sweet potato juice were significantly improved. Glu and Asp, with TAVs exceeding 1, were identified as significant ingredients to the sour/umami taste profile of sweet potato juice.

**Table 1:** Free amino acid concentration and TAV in sweet potato juice fermented with different LAB.

Taste^a^	Free amino acid	Threshold value^a^ (mg/kg)	Concentration (mg/kg)						TAV					
			CK	Lp10	Lc	St-Ld	Lr	Lp01	CK	Lp10	Lc	St-Ld	Lr	Lp01
Sour/Umami	Aspartate (Asp)	30	130.00 ± 2.00^c^	160.00 ± 3.00^a^	120.00 ± 2.00^d^	131.00 ± 2.00^c^	120.00 ± 2.00^d^	150.00 ± 2.00^b^	4.333	5.333	4	4.367	4	5
	Glutamate (Glu)	50	18.00 ± 1.00^d^	19.00 ± 1.00^d^	150.00 ± 3.00^b^	150.00 ± 2.00^b^	140.00 ± 1.00^c^	170.00 ± 3.00^a^	0.36	0.38	3	3	2.8	3.4
Total content			148.00^f^	179.00^e^	270.00^c^	281.00^b^	260.00^d^	320.00^a^	4.693	5.713	7	7.367	6.8	8.4
percentages/%			44.28	52.03	72.13	71.21	77.15	73.18	94.69	96.3	98.21	98.18	99.23	98.31
Sweet	Threonine (Thr)	2600	24.00 ± 0.50^a^	23.00 ± 1.00^ab^	20.00 ± 1.50^d^	21.00 ± 1.00^cd^	13.00 ± 0.50^e^	22.00 ± 0.10^bc^	0.009	0.009	0.008	0.008	0.005	0.008
	Serine (Ser)	1500	70.00 ± 2.00^b^	76.00 ± 2.00^a^	60.00 ± 3.00^d^	64.00 ± 2.40^c^	57.00 ± 2.00^e^	70.00 ± 1.00^b^	0.047	0.051	0.04	0.043	0.038	0.047
	Glycine (Gly)	1300	3.50 ± 0.05^b^	ND	ND	3.50 ± 0.01^b^	4.50 ± 0.01^a^	ND	0.003	–	–	0.003	0.003	–
	Alanine (Ala)	600	49.00 ± 1.00^a^	39.000 ± 1.00^b^	ND	ND	ND	ND	0.082	0.065	–	–	–	–
Total content			146.50^a^	138.00^b^	80.00^e^	88.50^d^	74.50^f^	92.00^c^	0.14	0.125	0.048	0.053	0.046	0.055
percentages/%			43.84	40.12	21.37	22.43	22.11	21.04	2.83	2.1	0.67	0.71	0.68	0.65
Bitter	Valine (Val)	400	21.00 ± 1.00^a^	16.00 ± 0.50^c^	11.00 ± 0.50^e^	17 ± 0.50^b^	2.50 ± 0.01^f^	15.00 ± 1.00^d^	0.053	0.04	0.028	0.043	0.006	0.038
	Lysine (Lys)	500	7.70 ± 0.10^a^	ND	4.60 ± 0.02^b^	ND	ND	ND	0.015	–	0.009	–	–	–
	Histidine (His)	200	11.00 ± 0.50^a^	11.00 ± 0.05^a^	8.70 ± 0.10^c^	8.1 ± 0.01^d^	ND	10.30 ± 0.10^b^	0.055	0.055	0.044	0.041	–	0.052
Total content			39.70^a^	27.00^b^	24.30^e^	25.10^d^	2.50^f^	25.3	0.14	0.125	0.048	0.053	0.046	0.055
percentages/%			11.88	7.85	6.49	6.36	0.74	5.79	2.48	1.6	1.13	1.11	0.09	1.04
Total free amino acid content			334.20^f^	344.00^d^	374.30^c^	394.60^b^	337.00^e^	437.30^a^	4.956	5.933	7.128	7.503	6.853	8.544

“-” means no data.

ND means not detect.

a Source: (Xiao et al., 2022); Data are mean ± standard deviation (SD) (*n* ≥ 3); Values within the same row with different letters are significantly different (*p* ≤ 0.05).

In comparison to CK, the Glu content in the fermented groups exhibited a marked increase, whereas the Asp content demonstrated minimal alterations without achieving statistical significance (*p* < 0.05). In terms of their influence on sour taste, Asp and Glu may possess a relatively minor effect compared to organic acids (Xiao et al., 2022), it was inferred that these two amino acids were also one of the sourness sources of fermented sweet potato juice. The other FAAs had no significant impact on the taste of fermented sweet potato juice, given that their TAVs were less than 1.

### 3.2 Analysis of VOCs

#### 3.2.1 VOCs Detected Utilizing HS-GC-IMS Technology

**Fig. 1:**
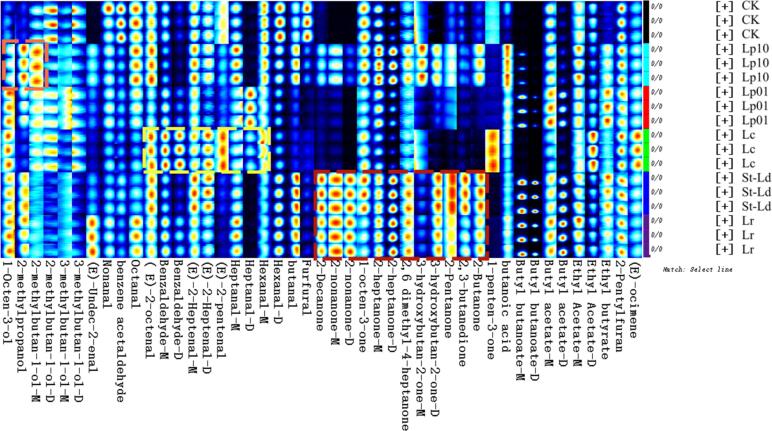
Fingerprints of VOCs in CK, Lp10, Lp01, Lc, St-Ld and Lr groups by HS-GC-IMS. The orange dashed rectangles, yellow dashed rectangles represent the VOCs with high signal strength in LP10 and Lc, respectively, and red dashed rectangle represents high signal intensity VOCs in St-Ld and Lr (Readers can refer to the web version of this article to understand the colors in this legend). (For interpretation of the references to color in this figure legend, the reader is referred to the web version of this article.)

Forty-five volatiles were identified by HS-GC-IMS, including 16 aldehydes, 13 ketones, 6 alcohols, 7 esters, 1 terpene, 1 furan, and 1 acid (Table S3). There was a significant difference in the fingerprint spectra between CK and fermented samples (Fig. 1).

After fermentation, the addition of butanoic acid, 3-hydroxybutan-2-one-M, 2-heptanone-M, heptanal-M (Fig. 1), and 1-octen-3-ol, 2-methylbutan-1-ol-M, 2-methylpropanol (Table S3) in Lp10 significantly increased the sour, green, butter, and fruit aroma in the sweet potato juice. Compounds including hexanal-M, heptanal-D, (*E*)-2-heptenal-D, benzaldehyde-D, benzaldehyde-M, (*E*)-2-octenal (Fig. 1), and 1-peten-3-one, ethyl acetate-D and (*E*)-ocimene (Table S3) were much higher in Lc group, and they contributed the flavor of lavender, green, fruit, cheesy, and fermented to Lc. The signal intensities of ketones compounds (except 1-penten-3-one) were higher in St-Ld and Lr groups, enhancing the flavor of sour, oringe, fresh, and fermented to St-Ld and Lr (Cadwallader & Singh, 2009). Compounds including ethyl butyrate, butyl butanoate-M and heptanal-D were higher in Lp01 group, contributing the flavor of fruit, apple, fatty and citrus to Lp01. After inoculation with LAB, the contents of ethyl butyrate, 2-pentyfuran, (*E*)-ocimene in each group significantly increased. The fermentation of Lr and St-Ld significantly increased the contents of ketones in fermented sweet potato juice. The aldehyde contents of Lc was higher than that of other groups.

Aldehydes were the greatest variety compounds detected and were mainly produced through the oxidation of unsaturated fatty acids and the hydrolysis of proteins during fermentation (Y. Q. Wang et al., 2020). During the fermentation process, the content of some aldehydes decreased while the content of alcohols increased, which might be related to the conversion of aldehydes into alcohols (Gao, Wang, Zhang, Sun, & Meng, 2022). There were four most abundant volatile compounds in sweet potato juices fermented with different LAB, which were 2-heptanone-D, butyl butanoate-D, 2-pentanone, and butyl butanoate-M. In a previous study on oxidative odors of dairy products, 2-heptanone was listed as the typical volatile components responsible for oxidative odors of soaps and dyestuff oxidized odors in dairy products (Cadwallader & Singh, 2009). According to the distribution and color of spots in the figure, the species and content of substances in CK, Lp10, Lc, St-Ld, Lr and Lp01 samples have changed significantly, and the species of substances in LAB fermented sweet potato juice are more abundant.

#### 3.2.2 VOCs Detected utilizing HS-SPME-GC–MS technology

According to Table S4, 47 VOCs were detected across all groups, encompassing 13 alcohols, 9 aldehydes, 9 ketones, 4 esters, 8 acids, 3 terpenes, and 1 phenol. Apparently, various LAB strains exerted notable impacts on the composition and concentration of VOCs.

According to the data presented in Table S4, a comprehensive analysis revealed the presence of 18 VOCs in the CK sample, with a cumulative concentration of 96.2 μg/kg. Furthermore, Lp10, Lp01, Lc, St-Ld, and Lr samples were examined, revealing 22, 22, 24, 23, and 27 VOCs, respectively. The total VOC content in these samples was 193.4, 255.3, 158.2, 129.7, and 272.1 μg/kg, respectively. These findings suggest a marked increase in the VOC composition and content following the inoculation of LAB.

**Fig. 2:**
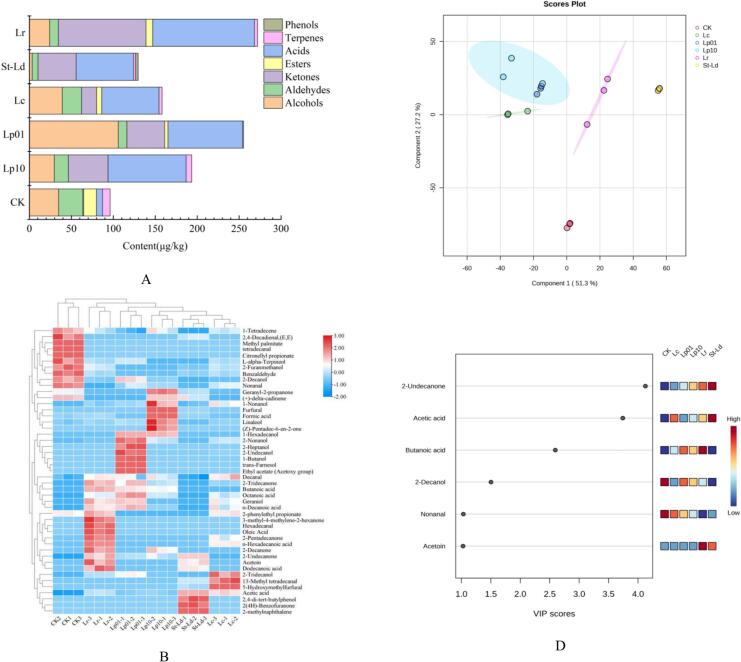
The multivariate statistical analysis of volatile organic compounds (VOCs) in sweet potato juice fermented with 5 LAB by GC–MS. (A) VOCs concentration histogram; (B) Hierarchical clustering heatmap; (C) Score scatter plot for PLS-DA model; (D) Differential compounds PLS-DA score plot of the GC–MS data. A VIP score close to or greater than 1 of the compound was considered to be a differential volatile in the cultivars.

Fig. 2B shows the visual information on the species and content of volatile compounds in the six different treatments of sweet potato juice. The contents of ketones and acids in the fermented sweet potato juice in Lp10, Lp01, Lc, St-Ld, Lr groups were significantly increased, while the aldehyde content was significantly decreased. Benzaldehyde, a compound with an attractive aroma, has sweet, nutty, and honey scents closely associated with a pleasant caramel and sweet aromas. Among the many aroma components of baked sweet potato, benzaldehyde is one of the most volatile components, which generated by the degradation process of aromatic amino acid phenylalanine (R. Zhang et al., 2021). (*E*,*E*)-2,4-Decadienal and 2-Furanmethanol were also major aldehyde components, which were regarded as aroma contributor to the baked sweet potato (Y. Wang & Kays, 2000).

A heatmap clustering analysis was conducted to gain a deeper understanding of the distinct differences among various strains on the flavor characteristics of fermented sweet potato juice (Fig. 2B). To intuitively understand the distribution of various volatile organic compounds, 47 VOCs were selected and their concentrations were used as variables. After normalization, the numerical range of each variable was unified, which was easy to compare and analyze. We used color intensity to represent the abundance of volatiles, in order of red and blue from high to low. Six groups were clustered into 4 categories, indicating that the inoculation of different LAB had a significant effect on the flavor of sweet potato juice.

Analysis of VOCs distribution was conducted using PLS-DA, with results presented in Fig. 2C. The plot depicting the PLS-DA scores clearly illustrates the model parameters of R^2^Y = 1, R^2^X = 0.9790, and Q^2^ = 0.961, indicating that the samples were distinctively separable. The variable importance in projection (VIP) score plot (Fig. 2D) revealed that six compounds—2-undecanone, acetic acid, butyric acid, 2-decanol, nonanal, and acetone—had VIP values exceeding 1. These findings suggest that these volatile compounds may serve as discriminatory volatiles for the five samples within the PLS-DA model.

#### 3.2.3 Comparative analysis of VOCs utilizing HS-GC-IMS and HS-SPME-GC–MS techniques

**Fig. 3:**
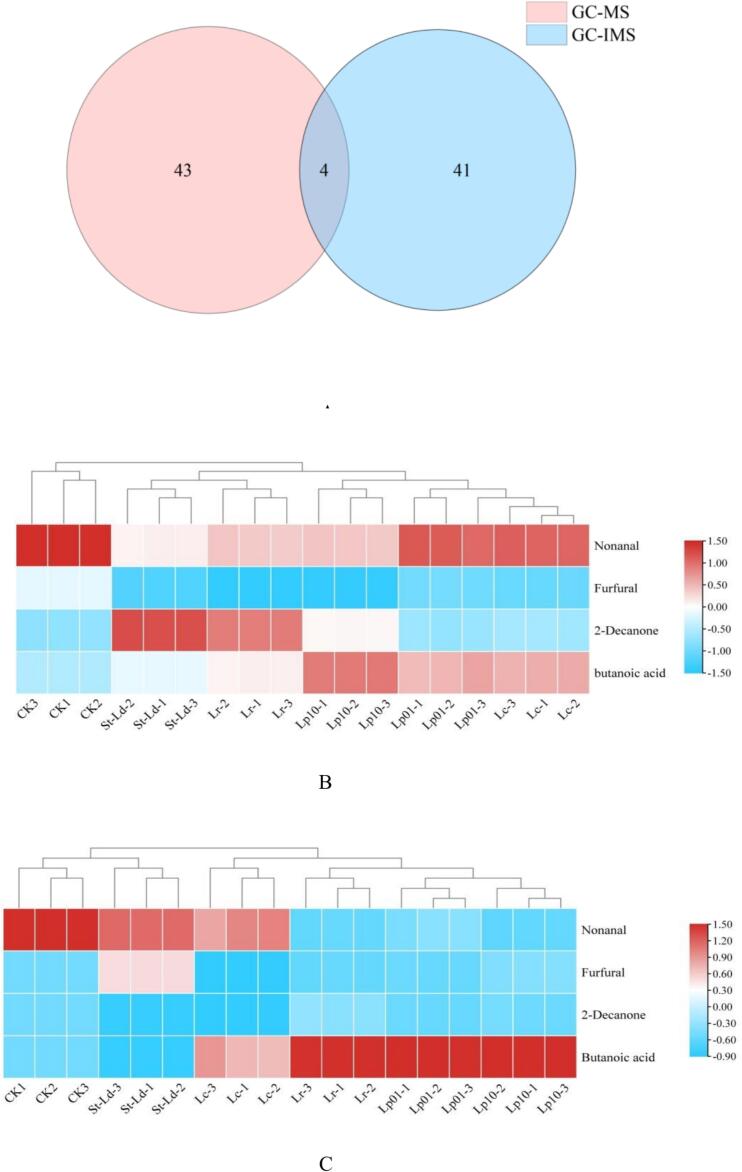
The multivariate statistical analysis of volatile organic compounds (VOCs) in sweet potato juice fermented with 5 LAB by GC–MS and GC-IMS. (A) Venn diagram of VOCs compared HS-GC-IMS with HS-SPME-GC–MS. (B) Changes in the content of mutual compounds identified by GC-IMS. (C) Changes in the content of mutual compounds identified by GC–MS.

To gain a deeper understanding of aroma profiles, we employed the techniques of HS-GC-IMS and HS-SPME-GC–MS to carry out an exhaustive analysis of VOCs within the CK, Lp10, Lp01, Lc, St Ld, and Lr groups. The experimental findings reveal notable disparities in the VOC recognition capabilities among the various groups, thereby offering robust evidence for the precise identification of aroma profiles. According to the results obtained from HS-SPME-GC–MS analysis, it was evident that acids, alcohols, and ketones constituted the primary volatile organic compounds (VOCs) present in the fermented sweet potato juice. However, HS-GC-IMS revealed elevated concentrations of aldehydes, ketones, and esters. It is noteworthy that phenols were exclusively detected by GC–MS, while furans were solely identified by GC-IMS. These observations underscore the distinct compound sensitivities exhibited by these two analytical methods. HS-GC-IMS successfully identified 45 VOCs, while HS-SPME-GC–MS detected 47 VOCs (Fig. 3). Notably, both methods detected four common compounds, and their respective alteration trends demonstrated significant consistency. This concordance underscores the value of combining HS-GC-IMS and GC–MS methodologies for a more comprehensive flavor profile analysis. Each method complements the other, providing a richer understanding of the volatile organic compounds present. Consequently, the integration of these two techniques offers a more comprehensive understanding of the flavor profiles, with both methods contributing complementary information.

### 3.3 Analysis of odor profiles and associated metabolic pathways in key aroma-active compounds (KACs)

Forty-eight VOCs were detected by GC–MS, but not all of them contributed had a significant impact to the flavor of fermented sweet potato juice. Therefore, the contribution of VOCs in fermented sweet potato juice to total aroma was estimated by calculating OAVs. Generally speaking, when OAV > 1, the compound was considered to be the KAC, contributing significantly to the overall aroma characteristics, and as the OAVs in the sample increases, the overall aroma of the compounds present increases proportionally (Yin et al., 2022). Based on OAV calculations, 8 VOCs (linalool, 1-nonanol, 2-undecanol, nonanal, decanal, 2-decanone, ethyl acetate, (+)-delta-cadinene) were identified as the KACs of fermented sweet potato juice.

Considering their lower OTs value and strong odor (including sweetness, fruitiness, floral aroma, and fat) characteristics, alcohols have been deemed to play a pivotal role in the olfactory perception of fermented sweet potato juice. Alcohols, a prevalent end-product in amino acid catabolism and glucose degradation (Cheng, 2010), is a crucial aromatic component in fermented sweet potato juice, which can give sweet potato juice an “elegant” aroma (Di Cagno, Filannino, & Gobbetti, 2017). As shown in Table S4, 13 alcohols were analyzed in the sweet potato juice. Based on the OAVs, linalool (sweet, fruity, floral), 1-nonanol (fresh, clean, fatty, floral), and 2-undecanol (waxy, fatty, clean, oily) were considered as the KACs of alcohols in the Lp01 group. For the rest of each fermentation group, linalool and 1-nonanol were considered KACs for Lp10, Lc and Lr, and only 1-nonanol was considered KAC for St-Ld. Alcohols usually have unique floral, sweet, or woody aromas, which have a good coordinating effect on the aroma of tea. Among them, linalool can be further synthesized through terpenoid main chain biosynthesis reactions. Lactobacillus have been reported in previous studies to produce linalool and impart a fruity flavor to fermented products(Li et al., 2023). *Lactobacillus* contains glycosylase, which hydrolyses glycosidic terpenes to produce linalool (Yang et al., 2023). The generation of 1-nonanol may be linked to unsaturated fatty acid degradation. According to reports, in the presence of copper based catalysts, 2-undecanol can be converted into aldehydes or ketones under mild conditions (Sun et al., 2023).

**Fig. 4:**
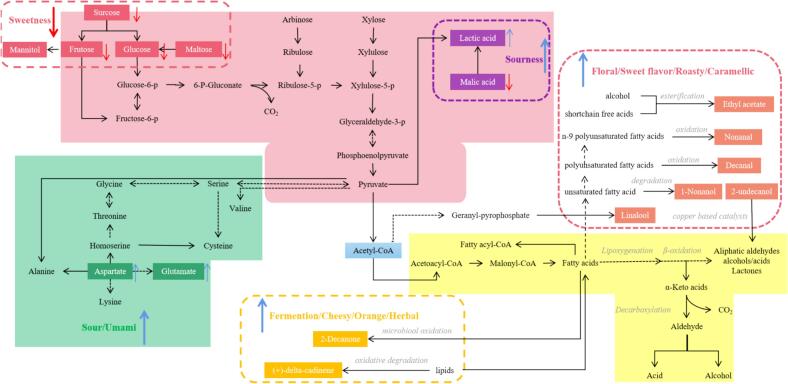
Possible metabolic pathways for flavor compounds of fermented sweet potato juice. The map was retrieved from the KEGG pathway database (https://www.genome. jp/kegg/pathway.html). The blue arrow represents an increase in the corresponding flavor, while the red arrow represents a decrease in the corresponding flavor.

Aldehydes are produced from the auto-oxidation and/or enzymatic oxidation of fatty acids (Ben-Harb et al., 2019). Nine aldehydes were identified in the sweet potato juice (Table S4). Based on the OAVs, decanal (fat, floral, fried, orange) was the KAC in all groups and impart fat, floral, fried, and orange aroma, while nonanal (fat, citrus) was the KAC in Lp10, Lp01, Lc and St-Ld. The generation of decanal may occur via the oxidation process of polyunsaturated fatty acids and nonanal is the product of the oxidation of n-9 polyunsaturated fatty acids (Fig. 4) (D. Zhao, Hu, & Chen, 2022).

Ketones possess a distinct flavor, even at moderate concentrations. Ziadi, Wathelet, Marlier, Hamdi, and Thonart (2008) found that the generation of ketones is primarily achieved through microbial oxidation of fatty acids or via decarboxylation pathways. This study found a total of 9 types of ketones in the samples. Based on the OAVs, 2-decanone (orange, floral, fatty, peach, fermented, cheesy) was the KAC of ketones in the Lp10 group and Lr group.

The generation of esters occurs through the esterification procedure, which entails the combination of short-chain free acids with alcohols. Four esters were identified in the sweet potato juice, as presented in Table S4. Based on the OAVs, ethyl acetate (fruity, sweet, weedy, green) was the KAC of ester in the Lp10 group, while no ester with OAV > 1 in other groups. Ethyl acetate, as a food seasoning agent, is widely used due to its fruity and sweet aroma (Alves et al., 2017).

Three terpenes were detected in the sweet potato juice as shown in Table S4. Hydrocarbons originate from the oxidative degradation of lipids, while branched hydrocarbons originate from the oxidation of branched aromatic acids (L. Zhang et al., 2022).

Furthermore, the concentrations and quantities of acids and phenol present in the samples were notably low, and they demonstrated elevated OTs, suggesting minimal alteration to the aroma profile of the fermented sweet potato juice.

In brief, in CK, there were 5 flavor compounds with an OAV exceeding 1, which are considered KACs for unfermented sweet potato juice, including 2 alcohols (total OAVs = 4.13), 2 aldehyde (total OAVs = 12.02), and 1 terpenes ((+)-delta-cadinene, OAV = 1.46), which imparted sweet, fruity, floral, fatty and thyme odor. Among them, the OAV value of nonanal was 10.44, which had the most vital aroma characteristic in CK. Eight flavor substances with OAV > 1 in Lp10, Lp01, Lc, St-Ld and Lr groups were considered as the KACs for fermented sweet potato juice, according to Table S4. After fermentation, the OAV of alcohols in all groups except St-Ld increased to varying degrees, the OAV of ketones and esters increased slightly, and the OAV of aldehydes and terpenes in all groups decreased to varying degrees. Besides alcohols, aldehydes and terpene were the primary aroma compounds responsible for imparting a fresh, floral, thyme, and citrus odor to the fermented sweet potato juice. As a result, inoculation fermentation by LAB reduced the aldehydes of sweet, fatty, and nutty odor, while adding more fresh, floral, thyme, and citrus aroma, thereby enriching the overall flavor and further enhancing the taste of sweet potato juice.

As shown in Fig. 4, based on KEGG analysis, possible metabolic pathways of KACs in LAB were thorough investigate, including glycolysis, pyruvate, amino acids, and lipids metabolism. The inoculation fermentation process carried out by LAB effectively reduced the sweet, fatty, and nutty odors in sweet potato juice, while significantly enhancing the freshness, floral, thyme, and citrus aroma profiles. The crucial role of LAB in shaping the flavor characteristics of fermented sweet potato juice was clearly evident.

### 3.4 Sensory evaluation

**Fig. 5:**
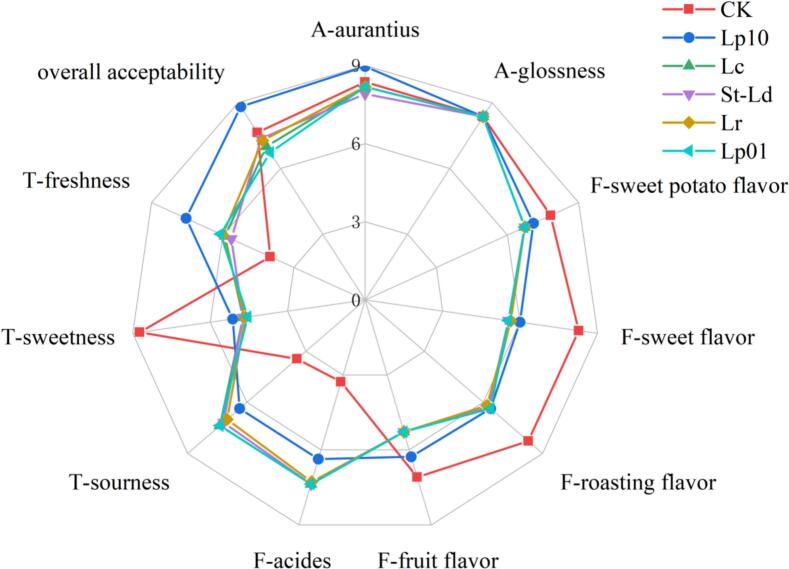
QDA profiles (Notes: A was appearance; F was flavor; T was taste).

As shown in Fig. 5, Lp10, Lp01, Lc, St-Ld and Lr groups displayed significantly higher intensities of sourness freshness and acids than CK, which was consistent with the results of organic acids and sugars analysis. In the evaluation of taste characteristics, the Lp10 group showed higher sourness and freshness compared with CK samples, which might result from the production of organic acids and mannitol during the heteromorphic fermentation process, a phenomenon that had a significant impact on the flavor and taste of food (Okoye et al., 2023). The sweetness of mannitol is equivalent to 61 % of the sweetness of sucrose, which has a cooling effect.

Lp10 group showed higher intensity of aurantius than other groups. Due to the absence of carbonyl groups in mannitol, it does not participate in the Maillard reaction and can reduce the browning of fruit juice (Antczak-Chrobot, Bak, & Wojtczak, 2017). The presence of mannitol can enrich the taste of LAB fermented sweet potato juices, giving them a cool taste and neutralizing the sweetness of sweet potatoes (Behare, Mazhar, Pennone, & McAuliffe, 2020). Lp10 group displayed lower intensities of sourness and acides than Lp01, Lc, St-Ld and Lr groups, which might be due to the difference of homotypic fermentation and heterotypic fermentation (Dicks & Endo, 2022). Five flavor characteristics were evaluated, fermentation groups displayed lower intensities of rosting flavor, fruit flavor, sweet flavor and sweet potato flavor than CK, acid was improved after fermentation. The glossness and aurantius between CK, Lp10, Lp01, Lc, St-Ld and Lr showed no obvious differences. The sweet potato juice fermented by inoculating Lp10 exhibited exceptional quality of flavor, appearance, and taste, thereby enhancing its overall acceptability.

With growing consumer demand for natural health beverages, fermented sweet potato juices are becoming increasingly popular due to their nutritional richness, unique flavor and potential health benefits. However, as these beverages evolve, they face new challenges in optimizing the fermentation process, regulating flavor metabolism and enhancing bioactivity.

While inoculated fermentation can regulate the fermentation process to some extent, it often results in homogenisation of flavor. Therefore, it is possible to develop a combination of strains for fermentation. This approach improves raw material utilization and shortens fermentation time, paving the way for a smarter and more automated production process. This integration is expected to merge the strengths of the two fermentation methods, resulting in the production of higher quality fermented juice.

## 4 Conclusion

This study investigated the effects of different LAB on the flavor characteristics of sweet potato juice, and achieved innovative research results, revealing the specific effects of LAB on sugars, organic acids, free amino acids content, and major aroma volatile metabolites. These findings have important guiding significance for a deeper understanding of the fermentation process of sweet potato juice and optimizing its flavor quality.

The findings revealed that the five LAB strains utilized in this study exhibited excellent growth potential in sweet potato juice. LAB reduced the sugar content, increased the levels of organic acids and amino acids, and further improved taste of sweet potato juice.LAB could also facilitate the production of VOCs, including acids and ketones, thereby enhancing the aroma quality. The sweet potato juice fermented by 5 strains had different flavor features, while the sample of Lp10 showed the highest overall acceptability. Lp10 could produce mannitol, which had given a more refreshing taste to the fermented sweet potato juice. Therefore, Lp10 (*L. plantarum* LD.0010) can be used as a potential starter culture for sweet potato juice.

## CRediT authorship contribution statement

**Bin Liang:** Writing – original draft. **Xue Bai:** Data curation. **Yunfan Wang:** Conceptualization. **Xiaohe Li:** Formal analysis. **Yanhui Kong:** Investigation. **Xiulian Li:** Visualization. **Xiangquan Zeng:** Supervision. **Wenli Liu:** Software. **Huamin Li:** Data curation. **Shuyang Sun:** Resources. **Hansheng Gong:** Supervision, Conceptualization. **Xinguang Fan:** Writing – review & editing, Visualization.

## Declaration of competing interest

We declare that we have no conflict of interest.

## Data Availability

Data will be made available on request.

## Appendix A Supplementary data

Supplementary material: Sugars, organic acids and VOCs of sweet potato juice fermented with different lactic acid bacteria. Image 1
