# Supplementary material for: Effect of five lactic acid bacteria on the flavor quality of fermented sweet potato juice
Source: Food Chem X. 2024 Nov 20;24:102023. doi: 10.1016/j.fochx.2024.102023 (PMC11626060; doi:10.1016/j.fochx.2024.102023)
Supplement: Supplementary file 1 — Supplementary material: Sugars, organic acids and VOCs of sweet potato juice fermented with different lactic acid bacteria. [file mmc1.docx]

Supplemental file

**Table S1.** Free sugars concentrations (mg/mL)in sweet potato juice fermented with different LAB at the end of fermentation

| Treatments | Content（mg/mL） | | | | |  |
| --- | --- | --- | --- | --- | --- | --- |
|  | Fructose | Mannitol | Glucose | Sucrose | Maltose | Total sugar |
| CK | 5.35±0.00^a^ | ^-^ | 6.56±0.00^a^ | 13.14±0.00^b^ | 30.18±0.00^a^ | 55.23±0.00^a^ |
| Lp10 | 0.14±0.00^f^ | 3.07±0.01^a^ | 2.44±0.01^d^ | 10.54±0.01^e^ | 12.82±0.01^e^ | 29.01±0.01^e^ |
| Lp01 | 3.17±0.01^d^ | ^-^ | 1.63±0.03^f^ | 12.14±0.03^c^ | 15.38±0.02^d^ | 32.32±0.03^d^ |
| Lc | 3.26±0.01^c^ | ^-^ | 2.36±0.01^e^ | 14.05±0.01^a^ | 19.55±0.02^c^ | 39.22±0.01^c^ |
| St-Ld | 3.43±0.01^b^ | ^-^ | 3.19±0.01^c^ | 10.32±0.01^f^ | 3.57±0.01^f^ | 20.51±0.01^f^ |
| Lr | 2.99±0.01^e^ | ^-^ | 3.85±0.02^b^ | 11.99±0.01^d^ | 20.37±0.10^b^ | 39.20±0.12^b^ |

All values are the mean ± SD.

Mean sharing different letters in the same column (a-f) are significantly different (*p* < 0.05).

**Table S2.** Organic acid concentrations (mg/mL) in sweet potato juice fermented with different LAB at the end of fermentation

| Treatments | Content (mg/L) | | | |  |
| --- | --- | --- | --- | --- | --- |
|  | Malic acid | Lactic acid | Citric acid | Fumaric acid | Total acid |
| CK | 270.17±7.57^a^ | 422.36±5.77^d^ | 429.96±5.33^a^ | 55.13±1.36^a^ | 1177.62±7.44^d^ |
| Lp10 | 116.27±13.07^b^ | 33,936.7±819.54^c^ | 235.27±10.42^b^ | 14.87±0.31^c^ | 34303.11±820.32^c^ |
| Lp01 | 119.53±7.84^b^ | 42,733.13±170.24^b^ | 246.7±10.06^b^ | 14.93±1.17^c^ | 43114.29±172.25^b^ |
| Lc | 112.93±13.74^b^ | 49,574.56±671.71^a^ | 239.43±8.62^b^ | 16.43±0.31^b^ | 49943.35±681.96^a^ |
| St-Ld | 115.50±13^b^ | 42,750.06±107.02^b^ | 242.77±6.67^b^ | 14.97±0.21^c^ | 43123.3±112.99^b^ |
| Lr | 118.77±8.45^b^ | 49,726.43±340.88^a^ | 244.37±10.55^b^ | 14.87±0.21^c^ | 50104.44±354.98^a^ |

All values are the mean ± SD.

Mean sharing different letters in the same column (a-d) are significantly different (*p* < 0.05).

| **Table S3.** The VOCs identified via HS-GC-IMS of sweet potato juice fermented with different lactic acid bacteria | | | | | | | | | | | | | | | |
| --- | --- | --- | --- | --- | --- | --- | --- | --- | --- | --- | --- | --- | --- | --- | --- |
| Number | Compounds | CAS | Formula | MW | RI | Rt [sec] | Dt [a.u.] | Comment | Volume of peak | | | | | | Order description |
|  |  |  |  |  |  |  |  |  | CK | Lp10 | Lp01 | Lc | St-Ld | Lr |  |
|  | Aldehydes（16） |  |  |  |  |  |  |  |  |  |  |  |  |  |  |
| 1 | (*E*)-Undec-2-enal | 53448-07-0 | C_11_H_20_O | 168.3 | 1512.1 | 1075.637 | 1.54853 |  | 123.49±12.58^f^ | 834.75±39.83^b^ | 531.7±10.93^d^ | 325.12±31.25^e^ | 611.10±33.94^c^ | 1,449.54±47.17^a^ | Fresh, fruity, citrus, orange peel |
| 2 | Nonanal | 124-19-6 | C_9_H_18_O | 142.2 | 1108.8 | 501.945 | 1.48049 |  | 885.25±23.62^a^ | 775.69±22.30^ab^ | 846.69±23.97^a^ | 613.98±56.23^b^ | 653.04±49.22^b^ | 751.01±45.12^ab^ | Fatty, citrus |
| 3 | Benzene acetaldehyde | 122-78-1 | C_8_H_8_O | 120.2 | 1040 | 404.026 | 1.2602 |  | 444.22±5.07^a^ | 222.36±12.58^d^ | 242.99±9.39^c^ | 228.49±10.75^cd^ | 278.22±1.96^b^ | 201.11±3.27^e^ | Rose, sweet |
| 4 | Octanal | 124-13-0 | C_8_H_16_O | 128.2 | 1005.2 | 354.543 | 1.4118 |  | 209.64±21.50^a^ | 223.14±10.88^a^ | 171.49±7.68^b^ | 121.59±15.16^c^ | 149.22±20.22^b^ | 219.42±11.21^a^ | Fatty |
| 5 | (*E*)-2-octenal | 2548-87-0 | C_8_H_14_O | 126.2 | 1056 | 426.826 | 1.33085 |  | 163.05±1.05^b^ | 178.63±4.44^a^ | 118.13±2.73^d^ | 175.83±6.96^a^ | 175.93±6.31^a^ | 149.30±1.84^c^ | Fatty, spicy |
| 6 | Benzaldehyde-M | 100-52-7 | C_7_H_6_O | 106.1 | 958.3 | 310.454 | 1.14872 | Monomer | 503.19±42.11^c^ | 325.83±0.51^e^ | 492.21±20.05^c^ | 707.87±42.45^a^ | 400.98±13.23^d^ | 622.14±11.81^b^ | Bitter, fruity |
| 7 | Benzaldehyde-D | 100-52-7 | C_7_H_6_O | 106.1 | 957.6 | 309.834 | 1.46819 | Dimer | 105.35±3.51^d^ | 65.23±1.28^e^ | 140.72±3.81^c^ | 373.42±10.67^a^ | 100.50±1.38^d^ | 201.95±9.38^b^ | Bitter, fruity |
| 8 | (*E*)-2-Heptenal-M | 18829-55-5 | C_7_H_12_O | 112.2 | 953.6 | 306.523 | 1.25648 | Monomer | 356.29±2.64^cd^ | 413.25±8.43^b^ | 349.46±14.63^d^ | 376.55±11.72^c^ | 451.87±13.18^a^ | 357.49±10.44^cd^ | Fatty |
| 9 | (*E*)-2-Heptenal-D | 29381-66-6 | C_7_H_12_O | 112.2 | 953.9 | 306.73 | 1.66849 | Dimer | 54.65±1.18^b^ | 57.32±5.42^b^ | 39.39±5.18^c^ | 75.23±7.96^a^ | 71.83±3.73^a^ | 50.53±2.55^b^ | Fatty |
| 10 | Heptanal-M | 111-71-7 | C_7_H_14_O | 114.2 | 899.1 | 261.203 | 1.32996 | Monomer | 122.36±4.71^c^ | 161.81±8.35^a^ | 135.49±6.64^bc^ | 138.97±4.44^b^ | 132.69±6.23^bc^ | 160.86±11.91^a^ | Fatty, citrus |
| 11 | Hexanal-D | 66-25-1 | C6H12O | 100.2 | 793 | 201.906 | 1.55744 | Dimer | 1,474.11±47.49^a^ | 861.07±11.89^c^ | 582.29±6.93^d^ | 583.68±40.70^d^ | 1,037.31±14.77^b^ | 1,072.08±10.91^b^ | Fruit, grass, green, cut grass, fresh |
| 12 | Heptanal-D | 111-71-7 | C_7_H_14_O | 114.2 | 900.5 | 262.368 | 1.69377 | Dimer | 37.28±1.26^c^ | 39.15±1.61^c^ | 468.66±6.75^a^ | 248.61±1.21^b^ | 33.13±9.45^c^ | 33.05±4.67^c^ | Fatty, citrus |
| 13 | Furfural | 98-01-1 | C5H4O2 | 96.1 | 827.4 | 220.441 | 1.08237 |  | 373.01±3.16^a^ | 168.55±5.12^b^ | 75.47±5.28^e^ | 80.83±5.91^e^ | 127.75±1.31^c^ | 102.81±4.65^d^ | Sweet, woody, bready, nutty, caramellic |
| 14 | Hexanal-M | 66-25-1 | C6H12O | 100.2 | 793 | 201.89 | 1.26015 | Monomer | 581.48±40.79 | 592.04±7.53 | 475.65±11.16^c^ | 630.66±66.88^a^ | 339.43±19.39^d^ | 376.02±3.42^d^ | Fruit, grass, green, cut grass, fresh |
| 15 | Butanal | 123-72-8 | C_4_H_8_O | 72.1 | 592.7 | 131.67 | 1.28847 |  | 405.68±8.81^c^ | 547.07±7.38^b^ | 200.56±11.36^d^ | 217.21±10.11^d^ | 580.72±8.92^a^ | 578.42±20.59^a^ | Chocolate |
| 16 | (*E*)-2-pentenal | 1576-87-0 | C5H8O | 84.1 | 749.7 | 183.443 | 1.10379 |  | 86.68±1.13^b^ | 62.98±3.91^c^ | 50.08±2.53^d^ | 112.00±5.36^a^ | 41.09±2.27^e^ | 33.16±2.71^f^ | Almond, caramel, cooked, roasted garlic, spice |
|  | Ktones(13) |  |  |  |  |  |  |  |  |  |  |  |  |  |  |
| 17 | 2-Decanone | 693-54-9 | C_10_H_20_O | 156.3 | 1251.8 | 705.383 | 1.47854 |  | 183.32±25.96^d^ | 659.56±19.37^c^ | 184.35±8.58^d^ | 191.80±29.65^d^ | 1,086.11±19.20^a^ | 939.32±2.62^b^ | Orange, floral, fattyty, peach, fermented, cheesy |
| 18 | 2-Nonanone-M | 821-55-6 | C_9_H_18_O | 142.2 | 1094.9 | 482.103 | 1.40668 | Monomer | 44.22±5.36^f^ | 1,097.98±14.47^c^ | 249.91±8.09^d^ | 195.40±10.62^e^ | 1,497.26±26.77^a^ | 1,431.9±60.35^b^ | Fresh, sweet, green,weedy, earthy,herbal |
| 19 | 2-Nonanone-D | 821-55-6 | C_9_H_18_O | 142.2 | 1094.7 | 481.819 | 1.88115 | Dimer | 42.63±3.15^c^ | 385.64±8.88^b^ | 46.62±3.97^c^ | 47.06±11.24^c^ | 1,257.73±10.32^a^ | 1,257.36±6.05^a^ | Fresh, sweet, green, weedy, earthy, herbal |
| 20 | 1-Octen-3-one | 4312-99-6 | C_8_H_14_O | 126.2 | 978.7 | 327.372 | 1.27524 |  | 171.13±5.19^bc^ | 179.99±7.11^b^ | 143.05±4.68^d^ | 158.92±6.58^c^ | 197.78±9.25^a^ | 128.36±8.81^e^ | Mushroom flavor |
| 21 | 2-Heptanone-M | 110-43-0 | C_7_H_14_O | 114.2 | 890.1 | 254.313 | 1.26007 | Monomer | 826.62±32.11^d^ | 1,506.08±26.84^a^ | 977.03±11.54^c^ | 727.56±68.84^e^ | 1,211.68±25.50^b^ | 1,232.77±44.77^b^ | Fruity, spicy |
| 22 | 2-Heptanone-D | 110-43-0 | C_7_H_14_O | 114.2 | 888.6 | 253.478 | 1.63144 | Dimer | 884.32±9.31^d^ | 3,972.3±15.39^c^ | 1421.38±42.71^b^ | 701.81±4.17^e^ | 4,365.11±23.43^b^ | 4,518.26±28.33^a^ | Fruity, spicy |
| 23 | 2,6 Dimethyl-4-heptanone | 108-83-8 | C_9_H_18_O | 142.2 | 951.5 | 304.757 | 1.32711 |  | 48.19±1.66^b^ | 71.16±1.28^a^ | 46.51±3.03^b^ | 50.61±7.12^b^ | 75.84±8.72^a^ | 69.39±1.12^a^ | Green, fermented fragrance |
| 24 | 3-Hydroxybutan-2-one-M | 513-86-0 | C4H8O2 | 88.1 | 712.5 | 168.675 | 1.05898 | Monomer | 211.28±18.23^bc^ | 519.1±26.81^a^ | 121.27±3.37^d^ | 234.26±26.25^b^ | 179.13±5.13^c^ | 184.11±21.26^c^ | Butter, cream, green pepper, rancid, sour |
| 25 | 3-Hydroxybutan-2-one-D | 513-86-0 | C4H8O2 | 88.1 | 712.2 | 168.558 | 1.32994 | Dimer | 142.44±2.11^f^ | 2,417.26±32.23^c^ | 680.75±4.55^d^ | 240.49±5.07^e^ | 3,153.66±56.39^a^ | 2,717.29±70.08^b^ | Butter, cream, green pepper, rancid, sour |
| 26 | 2-Pentanone | 107-87-9 | C_5_H_10_O | 86.1 | 681.7 | 157.209 | 1.36853 |  | 829.30±6.39^e^ | 2,196.73±24.02^c^ | 1,581.43±8.89^d^ | 240.92±13.59^f^ | 3,226.40±27.56^a^ | 2,700.88±22.28^b^ | Fruit |
| 27 | 2,3-Butanedione | 431-03-8 | C4H6O2 | 86.1 | 575.3 | 126.676 | 1.17401 |  | 542.48±8.59^d^ | 799.37±53.53^c^ | 347.57±18.78^e^ | 265.70±23.21^f^ | 2,079.68±24.12^a^ | 1,261.24±73.71^b^ | Butter, caramel, cheese, cream, fruit |
| 28 | 2-Butanone | 78-93-3 | C_4_H_8_O | 72.1 | 576.4 | 127.004 | 1.24501 |  | 164.97±3.03^c^ | 308.38±2.88^b^ | 114.07±7.46^d^ | 92.03±1.11^e^ | 404.10±9.33^a^ | 306.55±14.22^b^ | Butter, cream, green pepper, rancid, sour |
| 29 | 1-Penten-3-one | 1629-58-9 | C5H8O | 84.1 | 680.5 | 156.861 | 1.07639 |  | 42.07±2.15^b^ | 33.03±4.73^c^ | 26.31±1.38^d^ | 83.93±5.21^a^ | 17.80±3.05^e^ | 15.22±1.76^e^ | Green, herb, metal, mustard, pungent |
|  | Alcohols(6) |  |  |  |  |  |  |  |  |  |  |  |  |  |  |
| 30 | 1-Octen-3-ol | 3191-86-4 | C_8_H_16_O | 128.2 | 982.8 | 330.768 | 1.15526 |  | 98.05±18.66^d^ | 188.86±1.87^c^ | 265.71±11.26^a^ | 184.27±11.56^c^ | 224.15±11.73^b^ | 260.97±6.84^a^ | Mushroom and lavender flavors |
| 31 | 2-Methylpropanol | 78-83-1 | C_4_H_10_O | 74.1 | 623.1 | 140.41 | 1.16843 |  | 120.11±8.23^c^ | 319.30±23.15^a^ | 195.17±10.24^b^ | 103.38±18.44^c^ | 359.86±34.94^a^ | 311.19±43.21^a^ | Aroma of black tea and green tea |
| 32 | 2-Methylbutan-1-ol-M | 137-32-6 | C5H12O | 88.1 | 735.1 | 177.664 | 1.23026 | Monomer | 37.26±3.22^b^ | 48.15±3.06^a^ | 40.74±1.64^b^ | 36.04±1.08^b^ | 21.22±1.66^c^ | 26.8±7.06^c^ | Banana, fusel oil, green, malt, medicine |
| 33 | 3-Methylbutan-1-ol-M | 123-51-3 | C5H12O | 88.1 | 732.2 | 176.508 | 1.24291 | Monomer | 104.91±6.51^b^ | 62.85±3.48^d^ | 155.08±4.21^a^ | 73.35±6.11^c^ | 62.14±2.84^d^ | 62.45±7.21^d^ | Fruit, floral, banana, cocoa, fusel |
| 34 | 2-Methylbutan-1-ol-D | 137-32-6 | C5H12O | 88.1 | 732.2 | 176.508 | 1.47371 | Dimer | 111.62±2.64^a^ | 78.06±6.94^c^ | 80.43±3.01^c^ | 92.57±6.55^b^ | 46.22±8.38^d^ | 53.27±1.56^d^ | Banana, fusel oil, green, malt, medicine |
| 35 | 3-Methylbutan-1-ol-D | 123-51-3 | C5H12O | 88.1 | 730.3 | 175.738 | 1.49163 | Dimer | 66.39±3.48^a^ | 51.69±3.58^b^ | 54.00±3.12^b^ | 61.19±2.36^a^ | 34.46±3.61^d^ | 42.74±1.54^c^ | Fruit, floral, banana, cocoa, fusel |
|  | Esters(7) |  |  |  |  |  |  |  |  |  |  |  |  |  |  |
| 36 | Butyl butanoate-M | 109-21-7 | C_8_H_16_O_2_ | 144.2 | 730.3 | 175.738 | 1.49163 | Monomer | 203.24±1.78^e^ | 907.37±21.27^d^ | 1,986.44±12.23^c^ | 252.63±1.01^e^ | 3,196.74±33.43^a^ | 2,834.44±54.04^b^ | Apple |
| 37 | Butyl butanoate-D | 109-21-7 | C_8_H_16_O_2_ | 144.2 | 997.7 | 343.871 | 1.82243 | Dimer | 361.45±34.21^e^ | 463.30±38.17^d^ | 1,522.69±90.31^c^ | 362.10±30.28^e^ | 5,635.28±35.84^a^ | 3,847.36±67.42^b^ | Apple |
| 38 | Butyl acetate-D | 123-86-4 | C6H12O2 | 116.2 | 804.2 | 207.961 | 1.62048 | Dimer | 75.59±3.45^e^ | 99.80±3.01^d^ | 270.17±15.54^c^ | 67.97±2.34^e^ | 370.97±5.13^b^ | 405.82±2.29^a^ | Sweet, apple, banana, glue, pungent |
| 39 | Butyl acetate-M | 123-86-4 | C6H12O2 | 116.2 | 804.9 | 208.344 | 1.23773 | Monomer | 145.58±6.31^c^ | 142.74±4.41^c^ | 177.45±6.27^a^ | 115.15±7.39^d^ | 143.67±5.68^c^ | 164.14±3.11^b^ | Sweet, apple, banana, glue, pungent |
| 40 | Ethyl Acetate-M | 141-78-6 | C4H8O2 | 88.1 | 602.6 | 134.524 | 1.09492 | Monomer | 477.33±11.62^c^ | 538.13±40.01^b^ | 516.08±0.93^bc^ | 521.01±9.17^bc^ | 536.74±21.82^b^ | 600.04±54.94^a^ | Fruit, grape, balsamic, contact glue, pineapple |
| 41 | Ethyl Acetate-D | 141-78-6 | C4H8O2 | 88.1 | 603.2 | 134.702 | 1.33592 | Dimer | 1,435.05±15.74^b^ | 970.72±13.19^e^ | 1,324.72±12.61^c^ | 2,888.92±13.31^a^ | 1,122.71±29.01^d^ | 1,289.04±35.92^c^ | Fruit, grape, balsamic, contact glue, pineapple |
| 42 | Ethyl butyrate | 105-54-4 | C6H12O2 | 116.2 | 793.1 | 201.934 | 1.20602 |  | 36.27±4.79^e^ | 69.61±3.18^c^ | 82.49±2.35^b^ | 55.44±5.39^d^ | 90.01±2.94^a^ | 73.80±1.58^c^ | Fruit, apple, banana, butter, green pineapple |
|  | Terpenes(1) |  |  |  |  |  |  |  |  |  |  |  |  |  |  |
| 43 | (*E*)-ocimene | 3779-61-1 | C_10_H_16_ | 136.2 | 1052.6 | 421.975 | 1.21899 |  | 105.22±3.32^e^ | 122.46±1.88^b^ | 118.39±3.92^bc^ | 185.26±8.94^a^ | 111.09±3.91^cd^ | 106.69±3.41^e^ | Lavender |
|  | Furans(1) |  |  |  |  |  |  |  |  |  |  |  |  |  |  |
| 44 | 2-Pentylfuran | 3777-69-3 | C_9_H_14_O | 138.2 | 994.8 | 340.755 | 1.25174 |  | 154.12±5.67^ab^ | 162.74±1.28^a^ | 153.20±4.85^ab^ | 161.91±6.33^a^ | 154.88±7.93^ab^ | 144.57±4.79^b^ | Fatty, citrus |
|  | Acids(1) |  |  |  |  |  |  |  |  |  |  |  |  |  |  |
| 45 | Butanoic acid | 107-92-6 | C_4_H_8_O_2_ | 88.1 | 777.3 | 194.415 | 1.16359 |  | 255.48±16.33^d^ | 941.13±22.42^a^ | 613.83±41.18^bc^ | 495.69±17.87^c^ | 543.20±9.98^bc^ | 660.38±41.18^b^ | Butter, cheese, must, rancid, sour |

Odor description^a^: Cited from the website: https://www.flavornet.org

All values are the mean ± SD.

Mean sharing different letters in the same row (a-f) are significantly different (*p* < 0.05).**Table S4.** The VOCs identified via HS-SPME-GC-MS of sweet potato juice fermented with different lactic acid bacteria

| Number | Compounds | CAS | Formula | Odor description^a^ | OT^b^ (μg/kg) | Concentration(μg/kg） | | | | | | OAV | | | | | |
| --- | --- | --- | --- | --- | --- | --- | --- | --- | --- | --- | --- | --- | --- | --- | --- | --- | --- |
|  |  |  |  |  |  | CK | Lp10 | Lp01 | Lc | St-Ld | Lr | CK | Lp10 | Lp01 | Lc | St-Ld | Lr |
|  | Alcohols(13) |  |  |  |  |  |  |  |  |  |  |  |  |  |  |  |  |
| 1 | 1-Butanol | 71-36-3 | C_4_H_10_O | Fusel oil, sweet balsam, whiskey | 38 | ND | ND | 11.45 ±0.29^a^ | ND | ND | ND | - | - | ＜1 | - | - | - |
| 2 | 2-Heptanol | 543-49-7 | C7H16O | Citrus | 94 | ND | ND | 0.80 ±0.11^a^ | ND | ND | ND | - | - | ＜1 | - | - | - |
| 3 | 1-Hexadecanol | 36653-82-4 | C16H34O | Fatty, floral | - | ND | 2.61 ±0.12^b^ | 2.76 ±0.03^a^ | ND | ND | ND | - | - | - | - | - | - |
| 4 | 2-Nonanol | 628-99-9 | C9H20O | Fruit, cucumber, pungent | 280 | ND | 7.21 ±1.48^b^ | 22.74 ±1.17^a^ | 7.20 ±0.69^b^ | ND | 1.55 ±0.19^c^ | - | ＜1 | ＜1 | ＜1 | - | ＜1 |
| 5 | Linalool | 78-70-6 | C10H18O | Sweet, fruity, floral | 0.22 | 0.62 ±0.32^bc^ | 7.98 ±1.85^a^ | 1.73 ±0.22^b^ | 1.08 ±0.02^bc^ | ND | 1.23 ±0.24^bc^ | 2.82 | 36.27 | 7.86 | 4.91 | - | 5.59 |
| 6 | 2-Decanol | 1120-06-5 | C10H22O | - | - | 17.59 ±1.79^a^ | 4.03 ±0.19^c^ | 13.54 ±1.40^b^ | 1.91 ±0.11^d^ | ND | 3.43 ±0.60^cd^ | - | - | - | - | - | - |
| 7 | 1-Nonanol | 143-08-8 | C9H20O | Fresh, clean, fatty, floral, rose-like | 0.9 | 1.18 ±0.01^c^ | 4.66 ±1.12^a^ | 2.06 ±0.10^c^ | 3.15 ±0.47^b^ | 1.36 ±0.20^c^ | 1.78 ±0.21^c^ | 1.31 | 5.18 | 2.29 | 3.5 | 1.51 | 1.98 |
| 8 | 2-Furanmethanol | 98-00-0 | C5H6O2 | Burnt, sweet, caramellic, brown | 2000 | 9.59 ±1.00^a^ | ND | ND | 2.78 ±0.41^b^ | 0.82 ±0.15^c^ | 3.43 ±0.60^b^ | - | - | - | - | - | - |
| 9 | L-Alpha-Terpineol | 10482-56-1 | C10H18O | Lilac, floral | - | 5.85 ±0.95^a^ | ND | 2.18 ±0.02^b^ | 1.64 ±0.19^b^ | ND | 1.57 ±0.22^b^ | - | - | - | - | - | - |
| 10 | 2-Undecanol | 1653-30-1 | C11H24O | Waxy, fatty, clean, oily, fresh, fishy, nut | 8.6 | ND | ND | 33.18 ±3.06^a^ | 1.26 ±0.00^b^ | 0.10 ±0.00^b^ | 0.37 ±0.00^b^ | - | - | 3.86 | ＜1 | ＜1 | ＜1 |
| 11 | 2-Tridecanol | 1653-31-2 | C13H28O | Fruit, sweet | - | ND | 2.37 ±0.09^d^ | 9.28 ±0.67^b^ | 16.18 ±2.42^a^ | 1.01 ±0.14^de^ | 5.45 ±0.17^c^ | - | - | - | - | - | - |
| 12 | Geraniol | 106-24-1 | C10H18O | Geranium, lemon peel, passion fruit, peach, rose | 7.5 | ND | 0.77 ±0.01^c^ | 5.27 ±0.16^a^ | 3.92 ±0.73^b^ | ND | 5.30 ±0.89^a^ | - | ＜1 | ＜1 | ＜1 | - | ＜1 |
| 13 | Trans-Farnesol | 106-28-5 | C15H26O | Muguet, floral, sweet, lily | - | ND | ND | 1.01 ±0.00^a^ | ND | ND | ND | - | - | - | - | - | - |
|  | Aldehydes(9) |  |  |  |  |  |  |  |  |  |  |  |  |  |  |  |  |
| 14 | Nonanal | 124-19-6 | C9H18O | Fat, citrus | 1 | 10.44 ±0.55^a^ | 3.11 ±0.00^c^ | 4.07 ±1.30^bc^ | 4.94 ±0.61^b^ | 0.81 ±0.00^d^ | ND | 10.44 | 3.11 | 4.07 | 4.94 | ＜1 | - |
| 15 | 13-Methyl tetradecanal | 75853-51-9 | C15H30O | - | - | ND | ND | ND | 2.42 ±0.37^a^ | 0.61 ±0.00^b^ | ND | - | - | - | - | - | - |
| 16 | Furfural | 98-01-1 | C5H4O2 | Brown, sweet, woody, bready, nutty, caramellic | - | ND | 7.04 ±0.00^a^ | ND | ND | 0.55 ±0.00^b^ | ND | - | - | - | - | - | - |
| 17 | Decanal | 112-31-2 | C10H20O | Fat, floral, fried, orange peel, penetrating | 2 | 3.15 ±0.60^c^ | 6.75 ±1.19^ab^ | 6.11 ±2.00^b^ | 8.34 ±1.04^a^ | 2.14 ±0.72^b^ | 7.59 ±0.27^ab^ | 1.58 | 3.38 | 3.06 | 4.17 | 1.07 | 3.8 |
| 18 | Benzaldehyde | 100-52-7 | C7H6O | Sweet, oily, almond, cherry, nutty and woody | 350 | 9.52 ±0.61^a^ | ND | ND | 1.96 ±0.40^b^ | 1.13 ±0.17^c^ | 1.94 ±0.45^b^ | ＜1 | - | - | ＜1 | - | ＜1 |
| 19 | (E,E)-2,4-Decadienal | 65909-91-3 | C10H16O | Seaweed | - | 3.15 ±0.60^a^ | ND | ND | ND | 0.95 ±0.15^b^ | ND | - | - | - | - | - | - |
| 20 | Hexadecanal | 629-80-1 | C16H32O | Cardboard | - | ND | ND | ND | ND | ND | 0.86 ±0.10^a^ | - | - | - | - | - | - |
| 21 | Tetradecanal | 124-25-4 | C14H28O | Fatty, lactonic, coconut, woody | - | 2.62 ±0.01^a^ | ND | ND | ND | ND | ND | - | - | - | - | - | - |
| 22 | 5-Hydroxymethylfurfural | 67-47-0 | C6H6O3 | Fatty,buttery,herbal | - | ND | ND | ND | 5.44 ±0.00^a^ | 0.73 ±0.23^b^ | ND | - | - | - | - | - | - |
|  | Ketones(9) |  |  |  |  |  |  |  |  |  |  |  |  |  |  |  |  |
| 23 | Acetoin | 53584-56-8 | C4H8O2 | Creamy | 40000 | ND | ND | ND | ND | 8.28 ±1.01^b^ | 12.75 ±3.00^a^ | - | - | - | - | - | - |
| 24 | 2-Decanone | 693-54-9 | C10H20O | Orange, floral, fatty, peach, fermented, cheesy | 3 | ND | 4.75 ±1.14^b^ | ND | ND | ND | 7.55 ±1.26^a^ | - | 1.58 | - | - | - | 2.52 |
| 25 | 2-Undecanone | 112-12-9 | C11H22O | Waxy, fruity, creamy, fatty, orris, floral | - | ND | 21.27 ±5.34^b^ | 18.73 ±1.08^b^ | 6.64 ±0.21^c^ | 35.78 ±0.00^a^ | 37.40 ±4.18^a^ | - | - | - | - | - | - |

| 26 | 3-Methyl-4-methylene-2-hexanone | 20690-71-5 | C8H14O | - | - | ND | ND | ND | ND | ND | 12.08 ±2.15^a^ | - | - | - | - | - | - |
| --- | --- | --- | --- | --- | --- | --- | --- | --- | --- | --- | --- | --- | --- | --- | --- | --- | --- |
| 26 | 3-Methyl-4-methylene-2-hexanone | 20690-71-5 | C8H14O | - | - | ND | ND | ND | ND | ND | 12.08 ±2.15^a^ | - | - | - | - | - | - |

| 27 | Geranyl-2-propanone | 3796-70-1 | C13H22O | Floral, fruity, green, tropical, pear, banana, ylang, fatty | - | 1.05 ±0.06^b^ | 2.37 ±0.09^a^ | ND | ND | 0.63 ±0.07^c^ | ND | - | - | - | - | - | - |
| --- | --- | --- | --- | --- | --- | --- | --- | --- | --- | --- | --- | --- | --- | --- | --- | --- | --- |
| 28 | 2-Tridecanone | 593-08-8 | C13H26O | Fatty, waxy, dairy, milky, coconut, nutty, herbal, earthy | 500 | ND | 13.41 ±1.58^b^ | 26.51 ±5.19^a^ | 9.41 ±0.87^b^ | ND | 30.55 ±2.64^a^ | - | ＜1 | ＜1 | ＜1 | - | ＜1 |
| 29 | (Z)-Pentadec-6-en-2-one | 64667-26-1 | C18H34O | - | - | ND | 5.50 ±1.62^a^ | ND | ND | ND | ND | - | - | - | - | - | - |
| 30 | 2-Pentadecanone | 2345-28-0 | C15H30O | Fatty, spicy, floral | - | ND | ND | ND | 1.81 ±0.59^b^ | ND | 4.29 ±0.35^a^ | - | - | - | - | - | - |
| 31 | 2(4H)-Benzofuranone | 81800-41-1 | C11H16O2 | - |  | ND | ND | ND | ND | 0.91 ±0.08^a^ | ND | - | - | - | - | - | - |
|  | Esters(4) |  |  |  |  |  |  |  |  |  |  |  |  |  |  |  |  |
| 32 | Ethyl acetate (Acetoxy group) | 141-78-6 | C4H8O2 | Fruity, sweet, weedy, green | 2.2 | ND | ND | 3.80 ±0.00 | ND | ND | ND | - | - | 1.73 | - | - | - |
| 33 | Citronellyl propionate | 141-14-0 | C13H24O2 | Floral, green, waxy, rosy and citrus with fruity nuances | - | 2.62 ±0.01^a^ | ND | ND | ND | ND | ND | - | - | - | - | - | - |
| 34 | Methyl palmitate | 112-39-0 | C17H34O2 | Oily, fatty, orris | 2000 | 6.62 ±0.01^a^ | ND | ND | ND | ND | ND | - | - | - | - | - | - |
| 35 | 2-Phenylethyl propionate | 122-70-3 | C11H14O2 | Floral, balsamic, green, honey, sweet, rose, tropical | - | 5.81 ±0.40^b^ | ND | ND | 6.19 ±0.28^b^ | ND | 7.90 ±0.89^a^ | - | - | - | - | - | - |
|  | Acids(8) |  |  |  |  |  |  |  |  |  |  |  |  |  |  |  |  |
| 36 | Acetic acid | 64-19-7 | C2H4O2 | Sour, vinegar, acid, fruit, pungent | 230 | 5.50 ±1.30^e^ | 27.50 ±2.69^d^ | 26.80 ±2.29^d^ | 56.11 ±2.68^b^ | 65.71 ±0.69^a^ | 36.70 ±1.41^c^ | ＜1 | ＜1 | ＜1 | ＜1 | ＜1 | ＜1 |
| 37 | Formic acid | 64-18-6 | CH2O2 | Acetic, astringent, fruity, mustard, bready, with a pyruvic acid nuance | 141000 | 1.95 ±0.52^c^ | 17.40 ±0.00^a^ | ND | ND | 1.95 ±0.00^c^ | 2.73 ±0.38^b^ | - | - | - | - | - | - |
| 38 | Butanoic acid | 107-92-6 | C4H8O2 | Butter, cheese, must, rancid, sour | 240000 | ND | 45.06 ±1.86^c^ | 55.07 ±2.65^b^ | 4.43 ±0.00^d^ | ND | 69.46 ±2.69^a^ | - | - | - | - | - | - |
| 39 | Octanoic acid | 124-07-2 | C8H16O2 | Fatty, woody | 190 | ND | 2.23 ±0.13^b^ | 4.74 ±0.31^a^ | 2.34 ±0.47^b^ | ND | 2.67 ±0.24^b^ | - | ＜1 | ＜1 | ＜1 | - | ＜1 |
| 40 | Oleic Acid | 112-80-1 | C18H30O2 | Fatty, vegetable oil with lard and tallow nuances of french fried potatoes | - | ND | ND | ND | ND | ND | 0.62 ±0.05^a^ | - | - | - | - | - | - |
| 41 | n-Decanoic acid | 334-48-5 | C10H20O2 | Sour, fatty, citrus, fruity | 130 | ND | 0.77 ±0.00^c^ | 2.42 ±0.17^a^ | 1.62 ±0.55^b^ | ND | 2.08 ±0.20^a^ | - | ＜1 | ＜1 | ＜1 | - | ＜1 |
| 42 | Dodecanoic acid | 143-07-7 | C12H24O2 | Fatty, coconut oil | 7200 | ND | ND | ND | ND | 0.40 ±0.06^b^ | 0.72 ±0.16^a^ | - | - | - | - | - | - |
| 43 | n-Hexadecanoic acid | 1957-10-3 | C16H32O2 | Creamy, fatty | 10000 | ND | ND | ND | 3.69 ±0.12^b^ | 0.25 ±0.16^c^ | 6.34 ±0.31^a^ | - | - | - | - | - | - |
|  | Terpenes(3) |  |  |  |  |  |  |  |  |  |  |  |  |  |  |  |  |
| 44 | 1-Tetradecene | 1120-36-1 | C14H28 | Mineral oil | 60 | 6.62 ±0.75^a^ | 4.40 ±1.11^b^ | 1.04 ±0.73^c^ | 3.71 ±0.31^b^ | 0.46 ±0.01^c^ | 3.71 ±0.94^b^ | ＜1 | ＜1 | ＜1 | ＜1 | ＜1 | ＜1 |
| 45 | 2-Methylnaphthalene | 91-57-6 | C11H10 | Sweet, floral, woody, oily, aromatic | 10 | ND | ND | ND | ND | 0.63 ±0.00^a^ | ND | - | - | - | - | ＜1 | - |
| 46 | (+)-Delta-cadinene | 483-76-1 | C15H24 | Thyme, herbal, woody | 1.5 | 2.33 ±0.11^a^ | 2.24 ±0.00^a^ | ND | ND | 1.43 ±0.53^b^ | ND | 1.55 | 1.49 | - | - | ＜1 | - |
|  | Phenols(1) |  |  |  |  |  |  |  |  |  |  |  |  |  |  |  |  |
| 47 | 2,4-Di-tert-butylphenol | 96-76-4 | C14H22O | - | 500 | ND | ND | ND | ND | 3.02±0.37^a^ | ND | - | - | - | - | ＜1 | - |

Odor description^a^: Cited from the website: <https://www.flavornet.org/>

OT^b^ was mainly obtained from the website: <https://www.vcf-online.nl/VcfHome.cfm>

All values are the mean ± SD

Mean sharing different letters in the same row (a-f) are significantly different (*p* < 0.05).
